# Qualitative and Quantitative Detection of *Chlamydophila pneumoniae* DNA in Cerebrospinal Fluid from Multiple Sclerosis Patients and Controls

**DOI:** 10.1371/journal.pone.0005200

**Published:** 2009-04-09

**Authors:** Yi-Wei Tang, Subramaniam Sriram, Haijing Li, Song-yi Yao, Shufang Meng, William M. Mitchell, Charles W. Stratton

**Affiliations:** 1 Department of Pathology, Vanderbilt University School of Medicine, Nashville, Tennessee, United States of America; 2 Department of Medicine, Vanderbilt University School of Medicine, Nashville, Tennessee, United States of America; 3 Department of Neurology, Vanderbilt University School of Medicine, Nashville, Tennessee, United States of America; 4 Department of Microbiology and Immunology, Vanderbilt University School of Medicine, Nashville, Tennessee, United States of America; University of Hong Kong, Hong Kong

## Abstract

A standardized molecular test for the detection of *Chlamydophila pneumoniae* DNA in cerebrospinal fluid (CSF) would assist the further assessment of the association of *C. pneumoniae* with multiple sclerosis (MS). We developed and validated a qualitative colorimetric microtiter plate-based PCR assay (PCR-EIA) and a real-time quantitative PCR assay (TaqMan) for detection of *C. pneumoniae* DNA in CSF specimens from MS patients and controls. Compared to a touchdown nested-PCR assay, the sensitivity, specificity, and concordance of the PCR-EIA assay were 88.5%, 93.2%, and 90.5%, respectively, on a total of 137 CSF specimens. PCR-EIA presented a significantly higher sensitivity in MS patients (p = 0.008) and a higher specificity in other neurological diseases (p = 0.018). Test reproducibility of the PCR-EIA assay was statistically related to the volumes of extract DNA included in the test (p = 0.033); a high volume, which was equivalent to 100 µl of CSF per reaction, yielded a concordance of 96.8% between two medical technologists running the test at different times. The TaqMan quantitative PCR assay detected 26 of 63 (41.3%) of positive CSF specimens that tested positive by both PCR-EIA and nested-PCR qualitative assays. None of the CSF specimens that were negative by the two qualitative PCR methods were detected by the TaqMan quantitative PCR. The PCR-EIA assay detected a minimum of 25 copies/ml *C. pneumoniae* DNA in plasmid-spiked CSF, which was at least 10 times more sensitive than TaqMan. These data indicated that the PCR-EIA assay possessed a sensitivity that was equal to the nested-PCR procedures for the detection of *C. pneumoniae* DNA in CSF. The TaqMan system may not be sensitive enough for diagnostic purposes due to the low *C. pneumoniae* copies existing in the majority of CSF specimens from MS patients.

## Introduction

*C. pneumoniae* is an obligate intracellular pathogen that was first described as a cause of respiratory tract infections in humans [1]. A number of studies have suggested that *C. pneumoniae* infections may be associated with multiple sclerosis (MS) while other studies have found no association; this association and these studies have been recently reviewed [2], [3]. It is clear from these reviews that a standardized protocol for the molecular detection of *C. pneumoniae* would assist investigators who wish to further assess the association of this pathogen with MS.

The detection of *C. pneumoniae* in clinical specimens has been widely performed using PCR-based nucleic acid amplification techniques [4], [5] since the culture of this pathogen requires a cell culture and is complicated [4], [6]. Such PCR-based assays have improved the diagnosis of *C. pneumoniae* infections significantly. However, comparisons of different PCR assays for *C. pneumoniae* have demonstrated performance issues [7], [8] such that standardization of the PCR assay has been recommended [9]. Moreover, the possibility of low *C. pneumoniae* copies in the CSF suggests the need for extremely sensitive PCR detection methods [10]. Several approaches for increasing the sensitivity of PCR assays in CSF, including specimen concentration by high-speed centrifugation and Southern blots [11], [12], efficient DNA extraction [12], as well as nested-PCR methods [4], [13], have been described. However, high-speed centrifuges needed for specimen concentration may be unavailable in the routine clinical microbiology laboratory, Southern blots are impractical in high volume clinical microbiology laboratories, and nested-PCR procedures have a risk for carryover contamination and have not been recommended for use in routine diagnostic procedures.

In the present study, we reported the development and evaluation of a qualitative colorimetric microtiter plate-based PCR assay (PCR-EIA) and a real-time quantitative PCR assay (TaqMan) for detection of *C. pneumoniae* DNA in CSF. We applied these assays to the detection of *C. pneumoniae* in MS and other neurologic diseases (OND) controls due to the controversy in this field [2], [3]. The PCR-EIA assay was as sensitive as a comparative nested-PCR and more sensitive than the TaqMan quantitative PCR assay and offers a method more suitable for clinical laboratories. The quantitative PCR results from the TaqMan assay demonstrate that *C. pneumoniae* often is present in low copy numbers in the CSF of MS patients; this assay thus would not be suitable for diagnostic purposes.

## Methods

### Objectives

The objective of this study was to develop a standardized molecular test for the detection of *C. pneumoniae* DNA in CSF that was suitable for use in a clinical microbiology laboratory. We developed a qualitative PCR-EIA and a quantitative TaqMan PCR assay for detection of *C. pneumoniae* DNA in CSF specimens. These two PCR assays were compared to the nested-PCR assay in order to determine their sensitivity, specificity, and concordance. Finally, we validated these two PCR assays by testing CSF specimens from patients who were diagnosed with MS or with OND.

### Participants

CSF specimens were selected from patients from the Vanderbilt University Medical Center who satisfied the Poser criteria for the diagnosis of clinically definite MS. Age- and sex-matched patients with OND in whom CSF was being obtained for diagnostic studies were recruited as controls. CSF specimens were collected through routine lumber puncture procedures, aliquoted into 0.5 ml freezing vials within 1 hour of collection, and stored at −70°C or below prior to testing. A pooled clinical CSF specimen, which was herpesvirus and *C. pneumoniae*-negative, was used for a spiking assay to determine assay analytical sensitivities [14].

### Description of Procedures

#### DNA extraction

Nucleic acid was extracted from either 0.2 ml (high sample volume) or 0.1 ml (low sample volume) of CSF by using IsoQuick (Orca Research, Inc., Bothell, Wash.) according to the manufacturer's instruction as previously described [15]. The low volume extracted DNA samples were resuspended in 50 µl (low DNA content); the high volume samples were resuspended in 25 µl (high DNA content) of water to provide a 4× difference in DNA content between the low and high volume extracted samples. Human β-actin gene was amplified as a “house-keeping” gene for each sample DNA extract to validate quality and normalize quantity [16].

#### Qualitative PCR

A PCR-EIA assay was performed to detect *C. pneumoniae* DNA according to the procedure published previously [14], [15]. In brief, 50 µl of a PCR mixture contained the following: 1× buffer, 1.5 mM MgCl_2_, 10% glycerol, 200 µM dATP, dCTP, and dGTP, 100 µM dTTP, 90 µM dUTP, 10 µM digoxigenin-11-dUTP (Roche Biochemicals, Indianapolis, IN), 1 µM each primer, 0.01 units/µl uracil *N*-glycosylase (UNG, Epicentre Technologies, Madison, WI), 0.025 units/µl AmpliTaq gold DNA polymerase (Applied Biosystems), and either 25 µl (high volume) or 5 µl (low volume) of specimen DNA extract. The reaction mixtures were placed in an ABI 9700 thermal cycler (Applied Biosystems) programmed for a three-step PCR procedure followed by an initial UNG activation as described previously [14], [15]. The primer set (Cpm1, 5′-TTA CTT AAA GAA ACG TTT GGT AGT TCA TTT-3′ and Cpm2, 5′-TAA ACA TTT GGG ATC GCT TTG AT-3′) was designed to amplify a 154-bp portion of the *C. pneumoniae* major outer membrane protein (MOMP) gene [17], [18]. The amplification cycling conditions were a 5 min degradation of the preamplified templates at 50°C and then 50 cycles of denaturation at 94°C for 30 s, annealing at 55°C for 30 s, and extension at 72°C for 45 s [14]. Amplification products were then identified by detecting digoxigenin-labeled PCR products with a PCR ELISA kit (Roche Biochemicals) as previously described [14], [15], [19]. A *C. pneumoniae*-specific 5′-biotinylated capture probe (Cpmp, 5′-ACT CCG AAT AAA CCA ACG AGA TTG AAC GC-3′) was applied to detect and confirm the amplification product. Output signal was measured at an optical density of 450 (OD_450_). A positive result was defined as an OD_450_–OD_490_ value greater than or equal to 0.1.

#### Quantitative PCR

A real-time quantitative PCR assay was performed on the 7700 ABI Prism Sequence Detector (Applied Biosystems Foster City, CA). Sequences of primers and probe are the same as the ones used for qualitative detection. The amplicon was generated by PCR and was subsequently cloned into the pCR2.1 vector (Invitrogen, Carlsbad, CA). Quantitative pre- and post-DNA extraction standard curves were achieved by using five 10-fold serial dilutions of a plasmid standard (pCR-MOMP) containing the primer-spanning region of the *C. pneumoniae* MOMP B gene [17], [18]; this curve was determined both pre- and post-DNA extraction. The plasmid standard DNA concentration was calibrated by spectrophotometry at 260 nm. The fluorophore probe was designed with a reporter dye, 6-carboxyfluorescein (FAM) and a quencher dye, 6-carboxytetramethylrhodomine (TAMRA), covalently linked to the 5′ and 3′ ends, respectively [20], [21]. An aliquot of either 25 µl (high DNA content) or 5 µl (low DNA content) of the extracted nucleic acid was added to TaqMan Universal PCR Master Mix (Applied Biosystems) containing 0.8 µM of each primer and 0.4 µM probe to reach a final volume of 50 µl. The TaqMan cycling conditions were a 2 min degradation of the preamplified templates at 50°C and then 40 cycles of denaturation at 95°C for 15 s and annealing and extension at 58°C for 60 s.

#### Nested-PCR

DNA amplification of the *C. pneumoniae* MOMP gene was done using a touchdown nested-PCR technique as described previously [22], [23]. Briefly, DNA was extracted from 300 µl of CSF using the phenol chloroform method and dissolved in 30 µl of Tris-EDTA buffer. The presence of *C. pneumoniae* was examined by PCR using two different sets of internal and external primers that are specific for the *C. pneumoniae* MOMP gene [17], [18]. Amplification of the outside product was done first using the “touchdown” technique and 2 µl of the first product was then subjected to a second amplification using internal primers. The PCR products were visualized by 1.5% agarose gel electrophoresis staining with ethidium bromide [22], [23].

### Ethics

This study protocol was approved by the Vanderbilt University Institutional Review Board. Individual participants in this study were required to give written informed consent. In addition, the research involved the study of existing diagnostic specimens in which the information was recorded by the Investigator in such a manner that subjects could not be identified, directly or through identifiers linked to the subjects. Moreover, these diagnostic specimens had been collected before the research project began.

### Statistical methods

Data were entered into Microsoft Excel for further analysis. Results obtained from the nested-PCR were used as the evaluation standard. Comparisons of sensitivity, specificity and concordance by χ^2^ test were performed with Epi Info™ software (version 6; Centers for Diseases Control and Prevention, Atlanta, Ga.). A p value of ≤0.05 was considered statistically significant.

## Results

A total of 137 CSF specimens were included in the study to validate the PCR-EIA assay by using the nested-PCR procedure as the reference. The sensitivity and specificity of the PCR-EIA assay were 88.5% and 93.2% respectively when a high sample volume (equivalent to 100 µl CSF per reaction) of the CSF specimen was used (Table 1). The agreement rate between the PCR-EIA and the nested-PCR assays was 90.5%. The sensitivity of the PCR-EIA assay was significantly higher in specimens collected from MS patients (92.8%) than those from OND (55.6%) (p = 0.008). In contrast, the specificity was significantly higher in specimens collected from OND patients (97.9%) than those from MS (72.7%) (p = 0.018).

**Table 1:** Sensitivity and specificity of qualitative PCR assays (PCR-EIA versus nested-PCR) for detection of *C. pneumoniae* DNA in CSF specimens.

Clinical diagnosis	Number tested	nested-PCR +		nested-PCR −		Sensitivity (%)	Specificity (%)	Concordance (%)
		PCR-EIA +	PCR-EIA −	PCR-EIA +	PCR-EIA −			
Multiple sclerosis (MS)	80	64	5	3	8	92.8	72.7	90.0
Other neurologic diseases (OND)	57	5	4	1	47	55.6	97.9	91.2
Total	137	69	9	4	55	88.5	93.2	90.5

Test reproducibility of the PCR-EIA assay was determined on 31 CSF specimens by two different laboratorians on both high DNA content specimens (100 µl CSF per reaction) and low DNA content specimens (20 µl CSF per reaction). With the high DNA content specimens, a test reproducibility of 96.8% was reached between two different laboratorians (Table 2), which was significantly higher than that in low volume (74.2%) (χ^2^_M-H_ = 4.53, p = 0.033). The reproducibility appears to be a function of copy number of organisms per unit volume of CSF and was not confounded by other variables (p>0.05).

**Table 2:** Test reproducibility of PCR-EIA is related to extract volumes included in the PCR reaction mixture.

Clinical diagnosis	Number tested	Number positive by PCR-EIA	High sample volume		Low sample volume	
			Number matched	Concordance (%)	Number matched	Concordance (%)
Multiple sclerosis (MS)	24	21	23	95.8	18	75.0
Other neurologic diseases (OND)	7	1	7	100.0	5	71.4
Total	31	22	30	96.8	23	74.2

**Figure 1:**
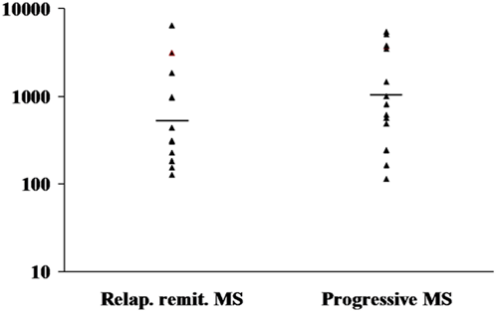
Dotplot of copy numbers of *C. pneumoniae* DNA in relapsing remitting MS (left) and progressive MS (right) patients. Horizontal bars indicated the geometric mean copy numbers, which were 567.7 and 1,009.6 copies/ml (geometric mean load) for relapsing remitting and progressive MS, respectively (p>0.05).

Quantitative standard curves were achieved by using five 10-fold dilutions of a plasmid standard containing the primer-spanning region of the *C. pneumoniae* MOMP gene covering plasmid copies from 2.5 to 25,000 per reaction, which corresponded to 25 to 250,000 copies/ml of CSF. A total of 79 CSF specimens were tested to validate the quantitative TaqMan assay. The TaqMan assay detected 26 specimens with a geometric *C. pneumoniae* mean load equivalent to 774.1 (115.2–6419.8) copies/ml of CSF (Table 3). All 26 positive specimens were from MS patients with positive results by both PCR-EIA and nested-PCR qualitative assays. TaqMan detected 12 (32.4%) of 37 CSF specimens from relapsing remitting MS patients in contrast to 14 (41.2%) of 34 specimens from progressive MS (p>0.05). *C. pneumoniae* loads measured by TaqMan were higher in CSF from progressive MS (geometric mean load±standard deviation: 1,009.6±3.6 copies/ml) than in CSF from relapsing remitting MS (567.7±3.6 copies/ml), but the difference was not statistically significant (Figure 1). None of the OND controls that were positive or negative by qualitative PCR were positive by TaqMan.

**Table 3:** *C. pneumoniae* loads in CSF specimens determined by TaqMan real-time quantitation.

Qualitative results^a^	Number tested	Number detected	Positive rate (%)
Positive	63	26^b^	41.3
Negative	8	0	0
Discrepancy^c^	8	0	0
Total	79	26	32.9

a Based on results obtained between PCR-EIA and nested-PCR assays.

b These 26 quantifiable specimens were all from MS patients with a geometric mean *C. pneumoniae* load equivalent to 774.1 (115.2–6419.8) copies/ml CSF.

c Included 5 nested-PCR+/PCR-EIA− and 3 nested-PCR−/PCR-EIA+ CSF specimens.

The post-DNA extraction test sensitivities of the PCR-EIA and TaqMan assays were determined by detecting six 10-fold dilutions of a plasmid standard covering plasmid copies from 0.025 to 2,500 per reaction. They were diluted using pooled *C. pneumoniae*-negative CSF specimens and processed as clinical specimens in triplicate, which corresponded to 0.25 to 25,000 copies/ml of CSF. All 6 reactions from 2 experiments were tested positive by the PCR-EIA assay for the dilution of 2.5 copies per reaction, indicating a test sensitivity of 25 copies/ml of CSF, which was at least 10 times more sensitive than the quantitative TaqMan assay (Table 4).

**Table 4:** Test sensitivity of *C. pneumoniae* DNA detection in plasmid-spiked CSF specimens.

Assay	Plasmid numbers per reaction (copies/ml CSF)						
	0	0.025 (0.25)	0.25 (2.5)	2.5 (25)	25 (250)	250 (2,500)	2,500 (25,000)
PCR-EIA	0/2	1/6^a^	3/6	6/6	6/6	6/6	6/6
TaqMan^b^	0/2	0/6	0/6	1/6	6/6	6/6	6/6

a Number positive/number detected.

b Positive results were defined as ≥25 copies/ml compared to standard curve generated by serially-diluted plasmid without spiking and extraction.

## Discussion

There is considerable controversy concerning the evidence for the presence of *C. pneumoniae* in CSF either from MS patients or from patients with other neurological diseases. The initial report of the association of *C. pneumoniae* and MS included both isolation of this microorganism by blinded cell cultures as well as the presence of *C. pneumoniae* DNA in CSF by PCR [11], [24]. A number of other investigators subsequently have confirmed the presence of *C. pneumoniae* DNA in CSF from MS patients by PCR methods, although usually in a smaller percent of patients [12], [25]–[30]. For example, Lay-Schmitt initially was able to show the presence of *C. pneumoniae* DNA in 22% of MS patients; this number increased to over 50% when phenol/chloroform extraction techniques were used [25]. A report by Geiffers found a high number of patients with other neurological diseases that demonstrated the presence of *C. pneumoniae* DNA in the CSF by PCR (21% for MS patients versus 43% for OND controls) [27]. This OND group included a number of individuals with inflammatory diseases including optic neuritis. Hence, these patients might represent patients with early symptoms of MS. In addition, several of these investigators have noted gene transcription of messenger RNA by *C. pneumoniae* in CSF from MS patients suggesting active infection by this pathogen [31], [32].

In contrast, other investigators have been unable to either isolate *C. pneumoniae* by cell culture [33], [34] or detect any chlamydial DNA in CSF from MS patients or controls [33]–[40] although one investigator was able to grow *C. pneumoniae* from one patient with MS despite negative PCR results in this patient [40]. In a collaborative study involving four different laboratories, the presence of *C. pneumoniae* was examined in 52 blinded CSF samples [41]. Dr. Sriram's laboratory at Vanderbilt was able to detect *C. pneumoniae* in 73% (22/30) of MS patients as compared to 22% (5/22) of controls. Three other laboratories (Drs. C. Gaydos, Johns Hopkins University, Baltimore MD; J. Boman, University Hospital, Umea Sweden; and L. Tondilla, CDC, Atlanta, GA) failed to show the presence of *C. pneumoniae* DNA in any of these CSF samples.

These inconsistencies of results from different laboratories strongly suggest that methodological problems are responsible. This is not surprising, as there are no well-accepted methods and techniques for demonstrating the presence of *C. pneumoniae* in CSF [9], [10]. Because of the difficulties in isolating *C. pneumoniae* in cell cultures, nucleic acid amplification methods such as PCR-based assays have become the method of choice for detection of this microorganism [5], [9]. However, PCR procedures often lack sensitivity, reproducibility, and specificity when applied to direct testing of clinical specimens [7], [8], [42]. This unsatisfactory performance of PCR appears to be related to the presence of inhibitors of the polymerase enzyme that are present in the clinical specimens, degradation of DNA, and contamination by DNA amplicons from previous testing. Other important issues related to PCR testing include the collection/storage of specimens, extraction of nucleic acids, target region and choice of primers, PCR method and reaction conditions, detection of amplification products, and the prevention and identification of false-positive and false-negative results.

The lack of standardized diagnostic methods for *C. pneumoniae* infection has resulted in controversy concerning the role of this microorganism in both atherosclerosis [43] and MS [2], [3]. In particular, PCR-based assays have been problematic. In a multicenter comparison of PCR-based assays for the detection of *C. pneumoniae* in endarterectomy specimens, the positivity rates varied between 0 and 60% among laboratories using different methods [44]. Similar technical problems may play an important role in the discrepant PCR results from CSF from MS patients tested in different laboratories. Ikejima and colleagues have demonstrated that PCR conditions must be optimized in order to obtain a signal for *C. pneumoniae* in the presence of low copy numbers of the organism [12]. For example, DNA extraction by the Qiagen QIAmp DNA Mini Kit with bacterial DNA extraction rather than the Qiagen QIAmp Blood Mini Kit has been shown to result in increased PCR sensitivity (20 copies versus 200 copies, respectively). The same investigators also showed that the primers for *C. pneumoniae* MOMP DNA gene had a 10-fold increase in sensitivity in comparison with primers for the 16s RNA gene. Smieja and colleagues have noted that replicate testing and the performance of probit analysis may be necessary to overcome the problems of dilution and statistical variability in the presence of low copy numbers [45]. Both Boman and Yamamoto have extensively reviewed many of the problems related to PCR assays [5], [10].

Microtiter plate-based PCR-enzyme linked amplification and hybridization assays for the detection of *C. pneumoniae* as well as other microorganisms have been described and are sensitive and robust methods suitable for the clinical laboratory [15], [46], [47]. The PCR-EIA assay as described in this report is simple, user-friendly, and results can be obtained in the same day. An additional antigen-antibody reaction was included as a signal amplification step to enhance test sensitivity. A recent blinded proficiency study indicated the PCR-EIA system detects as few as 1 copy/reaction human herpesvirus-6 in spiked plasma specimens [47]. The test sensitivity of the PCR-EIA in mock CSF samples is 25 *C. pneumoniae* organisms per ml of CSF. This is comparable to the sensitivity in mock CSF samples of the PCR assay described by Ikejima et al [12], which had a sensitivity of 100 organisms per ml of CSF. The nested-PCR assay used in this study has been shown previously to be as sensitive as the PCR assay described by Ikejima [12]. A uracil-*N*-glycosylase-based inactivation system was adapted to control for possible amplicon carryover contamination [14], [15], [19].

Several investigators have reported real-time PCR-based fluorescence assays for detection of *C. pneumoniae* [48], [49]. The TaqMan system described in this report incorporated a real-time format in which amplification and detection are accomplished simultaneously. This method measured the accumulation of PCR products using a fluorogenic probe and real-time laser scanning in a 96-well plate, and avoided potential carryover contamination by using a “closed” system [20], [21]. In this study, real-time quantitative PCR was not as sensitive as the PCR-EIA assay and therefore is not recommended for initial diagnostic purposes in CSF specimens. It could be useful, however, as a determinant of *C. pneumoniae* load and response to therapy in those cases with relatively high CSF content.

The sensitivity of the PCR-EIA assay was significantly higher in specimens collected from MS patients (92.8%) than those from OND (55.6%) (p = 0.008). In contrast, the specificity was significantly higher in specimens collected from OND patients (97.9%) than those from MS (72.7%) (p = 0.018). We believe that this difference may be due to the fact that although *C. pneumoniae* may be present in the CSF in a variety of neurological diseases and not restricted to multiple sclerosis [27], this pathogen is more likely to be found in the CSF from multiple sclerosis because the proinflammatory mediators produced by chronic intracellular chlamydial infections of ependymal cells located in the circumventricular organ [50] may produce a toxic effect on nearby mature CNS oligodendrocytes [51] and thus lead to demyelination and a diagnosis of multiple sclerosis.

This study did not detect significant differences in CSF *C. pneumoniae* loads between patients in different phases of MS. Although the *C. pneumoniae* loads were higher in CSF from progressive MS than that in relapsing remitting MS, the difference in geometric mean copy numbers was not statistically different.

This study has several key limitations. The first one is that the presence of DNA in the CSF does not mean that infection is playing a role in MS. This argues for the use of PCR-EIA to detect heat shock protein 60 (Hsp-60) mRNA from the CSF of patients with MS as was done by both Dong-Si et al [31] and Contini et al [32]. Hsp-60 is produced by *C. pneumoniae* during its cryptic phase. This study is being planned. A second one is that MS may not have a single infectious cause [2] or may involve an infectious trigger followed by an autoimmune response [3]. A sensitive PCR assay for Hsp-60 mRNA would also, in part, address this limitation. The final key limitation is that a nested-PCR assay was used as the validation reference since there is no “gold standard” technique available yet in the field. It is important to recognize the limitations of any diagnostic techniques used in the clinical laboratory.

In summary, the results of a quantitative PCR assay demonstrate that some CSF samples from MS patients have a low number of *C. pneumoniae* present. This confirms the argument that an extremely sensitive PCR assay is needed [10]. Nested-PCR methods have been used successfully, but nested procedures are not ideal for use in a clinical laboratory. The PCR-EIA assay described in this study is, however, more suited for a clinical laboratory and should allow accurate and sensitive detection of *C. pneumoniae* DNA in CSF.

## References

[1] Grayston JT, Campbell LA, Kuo CC, Mordhorst CH, P. Saikku P (1990). A new respiratory tract pathogen: *Chlamydia pneumoniae* strain TWAR.. J Infect Dis.

[2] Stratton CW, Wheldon DB (2006). Multiple sclerosis: an infectious syndrome involving *Chlamydophila pneumoniae*.. Trends Microbiol.

[3] Fainardi E, Castellazzi M, Seraceni S, Granieri E, Contini C (2008). Under the microscope: focus on *Chlamydia pneumoniae* infection and multiple sclerosis.. Curr Neurovasc Res.

[4] Boman J, Allard A, Persson K, Lundborg M, Juto P (1997). Rapid diagnosis of respiratory *Chlamdyia pneumoniae* infection by nested touchdown polymerase chain reaction compared with culture and antigen detection by EIA.. J Infect Dis.

[5] Boman J, Gaydos CA, Quinn TC (1999). Molecular diagnosis of *Chlamydia pneumoniae* infection.. J Clin Microbiol.

[6] Yan Y, Silvennoinen-Kassinen S, Leinonen M, Saikku P (2005). Methodological aspects affecting the infectivity of *Chlamydia pneumoniae* in cell cultures in vitro.. J Microbiol Methods.

[7] Mahony JB, Chong S, Coombes BK, Smieja M, Petrich A (2000). Analytical sensitivity, reproducibility of results, and clinical performance of five PCR assays for detecting *Chlamydia pneumoniae* DNA in peripheral blood mononuclear cells.. J Clin Microbiol.

[8] Fukano H (2004). Comparison of five PCR assays for detecting *Chlamydia pneumoniae* DNA.. Microbiol Immunol.

[9] Dowell SF, Peeling RW, Boman J, Carlone GM, Fields BS (2001). Standardizing Chlamydia pneumoniae assays: recommendations from the Centers for Diseases Control and Prevention (USA) and the Laboratory Centre for Disease Control (Canada).. Clin Infect Dis.

[10] Yamamoto Y (2002). PCR in diagnosis of infection: detection of bacteria in cerebrospinal fluids.. Clin Diagn Lab Immunol.

[11] Sriram S, Stratton CW, Yao S, Tharp A, Ding L (1999). *Chlamydia pneumoniae* infection of the central nervous system in multiple sclerosis.. Ann Neurol.

[12] Ikejima H, Haranaga S, Takemura H, Kamo T, Takahashi Y (2001). PCR-based method for isolation and detection of *Chlamdyia pneumoniae* DNA in cerebrospinal fluids.. Clin Diagn Lab Immunol.

[13] Black CM, Fields PI, Messmer TO, Berdal BP (1994). Detection of *Chlamydia pneumoniae* in clinical specimens by polymerase chain reaction using nested primers.. Eur J Clin Microbiol Infect Dis.

[14] Smalling TW, Sefers SE, Li HJ, Tang YW (2002). Molecular approaches to detecting herpes simplex and enteroviruses in the central nervous system.. J Clin Microbiol.

[15] Tang YW, Rys PN, Rutledge BJ, Mitchell PS, Smith TF (1998). Comparative evaluation of colometric microtiter plate systems for detection of herpes simplex virus in cerebrospinal fluid.. J Clin Microbiol.

[16] Ng SY, Gunning P, Eddy R, Ponte P, Leavitt J (1985). Evolution of the functional human beta-actin gene and its multi-pseudogene family: conservation of noncoding regions and chromosomal dispersion of pseudogenes.. Mol Cell Biol.

[17] Melgosa P, Kuo CC, Campbell LA (1991). Sequence analysis of the major outer membrane protein gene of *Chlamydia pneumoniae*.. Infect Immun.

[18] Watson MW, Lambden PR, Clarke IN (1991). Genetic diversity and identification of human infection by amplification of the chlamydial 60-kilodalton cysteine-rich outer membrane protein gene.. J Clin Microbiol.

[19] Tang YW, Mitchell PS, Espy MJ, Smith TF, Persing DH (1999). Molecular diagnosis of herpes simplex virus infections in the central nervous system.. J Clin Microbiol.

[20] Holland PM, Abramson RD, Watson R, Gelfand DH (1991). Detection of specific polymerase chain reaction product by utilizing the 5′-3′ exonuclease activity of *Thermus aquaticus* DNA polymerase.. Proc Natl Acad Sci USA.

[21] Heid CA, Stevens J, Livak KJ, Williams PM (1996). Real time quantitative PCR.. Genome Res.

[22] Khan MA, Potter CW (1996). The nPCR detection of *Chlamydia pneumoniae* and *Chlamydia trachomatis* in children hospitalized for bronchiolitis.. J Infect.

[23] Sriram S, Yao SY, Stratton C, Calabresi P, Mitchell WM (2002). Comparative study of the presence of *Chlamydia pneumoniae* in cerebral spinal fluid from clinically definite and monosymptomatic multiple sclerosis.. Clin Diagn Lab Immunol.

[24] Sriram S, Mitchell W, Stratton C (1998). Multiple sclerosis associated with *Chlamydia pneumoniae* infection of the CNS.. Neurology.

[25] Layh-Schmitt G, Bendi C, Hildt U, Dong-Si T, Juttler E (2000). Evidence for infection with *Chlamydia pneumoniae* in a subgroup of patients with multiple sclerosis.. Ann Neurol.

[26] Sotgiu S, Piana A, Pugliatti M, Sotgiu A, Deiana GA (2001). *Chlamydia pneumoniae* in the cerebrospinal fluid of patients with multiple sclerosis and neurological controls.. Mult Scler.

[27] Gieffers J, Pohl D, Treib J, Dittmann R, Stephan C (2001). Presence of *Chlamydia pneumoniae* DNA in the cerebral spinal fluid is a common phenomenon in a variety of neurological diseases and not restricted to multiple sclerosis.. Ann Neurol.

[28] Hao Q, Miyashita N, Wang H-Y, Matsushima T, Saida T (2002). *Chlamydia pneumoniae* infection associated with enhanced MRI spinal lesions in multiple sclerosis.. Mult Scler.

[29] Grimaldi LM, Pincherle A, Martinelli-Boneschi F, Fillippi M, Patti F (2003). An MRI study of *Chlamydia pneumoniae* infection in Italian multiple sclerosis patients.. Mult Scler.

[30] Contini C, Cultrera R, Seraceni S, Castellazzi M, Granieri E (2004). Cerebrospinal fluid molecular demonstration of *Chlamydia pneumoniae* DNA is associated to clinical and brain magnetic resonance imaging activity in a subset of patients with relapsing-remitting multiple sclerosis.. Mult Scler.

[31] Dong-Si T, Weber J, Liu YB, Buhmann C, Bauer H (2004). Increased prevalence of and gene transcription by *Chlamydia pneumoniae* in cerebrospinal fluid of patients with relapsing-remitting multiple sclerosis.. J Neurol.

[32] Contini C, Seraceni S, Castellazzi M, Granieri E, Fainaardi E (2008). *Chlamydophila pneumoniae* DNA and mRNA transcript levels in peripheral blood mononuclear cells and cerebrospinal fluid of patients with multiple sclerosis.. Neurosci Res.

[33] Boman J, Roblin PM, Sundstrom P, Sandstrom M, Hammerschlag MR (2000). Failure to detect *Chlamydia pneumoniae* in the central nervous system of patients with MS.. Neurology.

[34] Hammerschlag MR, Ke Z, Lu F, Roblin P, Boman J, Kalman B (2000). Is *Chlamydia pneumoniae* present in brain lesions of patients with multiple sclerosis?. J Clin Microbiol.

[35] Morre SA, De Groot CJ, Killestein J, Meijer CJ, Polman CH (2000). Is *Chlamydia pneumoniae* present in the central nervous system of multiple sclerosis patients?. Ann Neurol.

[36] Pucci E, Taus C, Cartechini E, Morelli M, Giuliani G (2000). Lack of *Chlamydia* infection of the central nervous system in multiple sclerosis.. Ann Neurol.

[37] Saiz A, Marcos MA, Graus F, Vidal J, Jimenez de Anta MT (2001). No evidence of CNS infection with *Chlamydia pneumoniae* in patients with multiple sclerosis.. J Neurol.

[38] Derfuss T, Gurkov R, Then Bergh F, Goebels N, Hartmann M (2001). Intrathecal antibody production against Chlamydia pneumoniae in multiple sclerosis is part of a polyspecific immune response.. Brain.

[39] Numazaki K, Chibar S (2002). Failure to detect *Chlamydia pneumoniae* in the central nervous system of patients with MS.. Neurology.

[40] Furrows SJ, Hartley JC, Bell J, Silver N, Losseff N (2004). *Chlamydophila pneumoniae* infection of the central nervous system in patients with multiple sclerosis.. J Neruol Neurosurg Psychiatry.

[41] Kaufman M, Gaydos CA, Sriram S, Boman J, Tondella ML (2002). Is *Chlamydia pneumoniae* found in spinal fluid samples from multiple sclerosis patients? Conflicting results.. Mult Scler.

[42] Fredricks DN, Relman DA (1999). Application of polymerase chain reaction to the diagnosis of infectious diseases.. Clin Infect Dis.

[43] Boman J, Hammerschlag MR (2002). *Chlamydia pneumoniae* and atherosclerosis: critical assessment of diagnostic methods and relevance to treatment studies.. Clin Microbiol Rev.

[44] Apfalter P, Blasi R, Boman J, Gaydos CA, Kundi M (2001). Multicenter comparison trial of DNA extraction methods and PCR assays for detection of *Chlamydia pneumoniae* in endarterectomy specimens.. J Clin Microbiol.

[45] Smieja M, Mahony JB, Goldsmith CH, Chong S, Petrich A (2001). Replicate PCR testing and probit analysis for detection and quantitation of *Chlamdyia pneumoniae* in clinical specimens.. J Clin Microbiol.

[46] Inman-Bamber J, Wan C, Gardam T, Vohra R, van Daal A (2002). Novel PCR-EIA method for the detection of *Chlamydia pneumoniae* in respiratory specimens.. Mol Cell Probes.

[47] Flamand L, Gravel A, Boutolleau D, Alvarez-Lafuente R (2008). Multicenter comparison of PCR assays for detection of human herpesvirus 6 DNA in serum.. J Clin Microiol.

[48] Kuoppa Y, Bowman J, Scott L, Kumlin U, Eriksson I (2002). Quantitative detection of respiratory *Chlamyidia pneumoniae* infection by real-time PCR.. J Clin Microbiol.

[49] Tondella ML, Talkington DF, Holloway BP, Dowell SF, Cowley K (2002). Development and evaluation of real-time PCR fluorescence assays for detection of *Chlamydia pneumoniae*.. J Clin Microbiol.

[50] Sriram S, Ljunggren-Rose A, Yao S-Y, Whetsell WO (2005). Detection of chlamydial bodies and antigens in the central nervous system of patients with multiple sclerosis.. J Infect Dis.

[51] Kong G-Y, Kristensson K, Bentivoglio M (2002). Reaction of mouse brain oligodendrocytes and their precursors, astrocytes and microglia, to proinflammatory medicators circulating in the cerebrospinal fluid.. GLIA.

